# Recruitment Maneuver in Elderly Patients with Different Peripheral Chemoreflex Sensitivity during Major Abdominal Surgery

**DOI:** 10.1155/2016/2974852

**Published:** 2016-12-13

**Authors:** Nikita Trembach, Igor Zabolotskikh

**Affiliations:** Kuban State Medical University, Russian Federation, Sedin str. 4, Krasnodar 350012, Russia

## Abstract

The goal of the study was to evaluate the effect of a recruitment maneuver on respiratory biomechanics, oxygenation, and hemodynamics in patients suffering from chronic heart failure with different peripheral chemoreflex sensitivity. The study was conducted in 115 elderly patients which underwent major abdominal surgery under general/epidural surgery. Peripheral chemoreflex sensitivity (PCS) was evaluated with breath-holding duration (BHD) during breath-holding test. All patients were divided into two groups: group H had a high PCS (BHD = 38 seconds or less, *n* = 49); Group M had a middle PCS (BHD more than 38 seconds, *n* = 66). Recruitment maneuver improved oxygenation and respiratory biomechanics in all cases. However, cardiac output decreased by an average of 18%–31% in group H compared to 18%–28% in group M. SVR either remained unchanged or decreased by up to 14% of the initial value in group H, while, in group M, it had a tendency to increase, which was 24% of the initial value. So, recruitment maneuver is an effective method to improve oxygenation and biomechanical properties of the respiratory system but in patients with increased peripheral chemoreflex sensitivity it associates with the risk of hemodynamic disturbances.

## 1. Introduction

Major abdominal surgery is accompanied by intraoperative mechanical ventilation, which is often associated with the development of respiratory disorders due to atelectasis formation [1]. The reason for this lies in the dysfunction of surfactant, resorption of alveolar gas, and mechanical compression of lung tissue [2]. Finally, atelectasis leads to a decrease in oxygenation and becomes a major cause of postoperative hypoxemia [1] and is likely to place the development of an infectious process, greatly increasing the risk of respiratory complications [3]. The “open lung” concept is widely used in practice to prevent these disorders. Basically it consists in the application of positive end-expiratory pressure (PEEP) to maintain end-expiratory lung volume, which significantly reduces the severity of atelectasis and improves oxygenation [4]. However, the maintenance of PEEP is often not enough, because it is difficult to set its proper level that anyway results in atelectasis formation [5]. A lung recruitment maneuver (RM) allows us to reexpend the collapsed alveoli and maintain oxygenation. Combined application of PEEP and RM leads to a reduction of postoperative pulmonary complications [6] that makes some authors include this combination into an algorithm for protective intraoperative ventilation [7]. However, such a strategy of mechanical ventilation may be associated with adverse hemodynamic effects associated with the increase in right ventricular afterload and increase in intrathoracic pressure, which ultimately leads to a decrease in cardiac output and blood pressure [8]. Elderly patients with chronic heart failure are at risk for postoperative respiratory complications and in this case the implementation of protective ventilation strategy is highly indicated, but, on the other hand, these patients are at risk of hemodynamic instability during positive-pressure ventilation and it is quite difficult to evaluate how the patient in this group will tolerate the RM.

A significant increase in the sensitivity of the peripheral chemoreflex was noted in severe chronic heart failure (CHF), unstable hypertension, and sleep apnea syndrome [9]. Mechanisms for increasing the chemoreflex sensitivity in chronic heart failure were studied in recent years especially carefully, due to the fact that the degree of the increase is directly correlated with the severity of cardiovascular disorders [10]. The pathogenesis of hypersensitivity in severe forms of heart failure is quite complex and not fully understood. The main mechanisms are nitric oxide synthase downregulation and impaired regulation of angiotensin [11], observed in CHF. Dysregulation of the effects of nitric oxide and angiotensin causes a change in level of functioning of potassium channels responsible for sensitivity to hypoxic stimulus in cells of the carotid glomus [12, 13]. Also, chronic cardiac disease is associated with morphological changes in the structures of the carotid glomus and decreased baroreflex sensitivity. In modern literature, there is also evidence of increasing the sensitivity of peripheral chemoreceptors to the hypoxic stimulus in patients with chronic obstructive pulmonary disease and patients with chronic kidney disease, but the mechanism of this phenomenon has not been studied. Moreover, changes in the reflex regulation of cardiorespiratory system in chronic diseases, particularly in CHF, involve a decreasing in arterial baroreflex sensitivity, and the degree of this decrease is inversely proportional to the sensitivity of the peripheral chemoreflex sensitivity [14] that ensures the maintenance of hemodynamics during its sudden changes [15]. Thus, PCS can be a predictor of hemodynamic instability and cardiovascular critical incidents during mechanical ventilation.

Thus, the goal of the study was to evaluate the effect of a recruitment maneuver on respiratory biomechanics, oxygenation, and hemodynamics in patients suffering from chronic heart failure with different peripheral chemoreflex sensitivity.

## 2. Materials and Methods

The study was conducted in 115 elderly patients (mean age 70 (66–75) years), which underwent elective major abdominal surgical interventions for cancer of the colon. Open hemicolectomy, resection, and extirpation of the rectum in supine position were performed in the patients. The average duration of operations was 4 ± 1 hours. All patients had ASA III physical status and CHF NYHA II functional class. Exclusion criteria were as follows: concomitant pulmonary pathology, history of thoracic surgery, significant systolic dysfunction (LVEF less than 40%), and body mass index over 35 kg/m^2^. All participants gave their informed consent to participate in this study that was previously approved by the Local Ethics Committee of Kuban State Medical University (Russian Federation, Krasnodar) in 02.02.2016.

The day before the surgery and prior to premedication, PCS was evaluated with a breath-holding duration (BHD) test. After inspiration of 2/3 of lung vital capacity and a voluntary breath-holding, apnea was measured from the beginning of the test till the emerging of reflex contractions of the diaphragm, determined by palpation.

All patients were divided into two groups: group H had a high PCS (BHD = 38 seconds or less, *n* = 49); Group M had middle PCS (BHD more than 38 seconds, *n* = 66). Such division is based on the results of works showing that in patients with CHF breath-holding duration of 38 seconds or less is associated with high risk of hemodynamic instability [16].

The induction of anesthesia was performed in all groups using the following drugs: propofol 2 mg/kg, fentanyl in a dose of 3 mg/kg, nondepolarizing relaxant, atracurium (0.5 mg/kg). Sevoflurane was used to maintain the anesthesia. The depth of anesthesia was monitored by bispectral index, which was maintained at the level of 40–60. Before induction the epidural space was catheterized in all patients in operating room with 18G Tuohy needle in Th10-Th11 level with the introduction of 40 mg of lidocaine as a test dose. Intraoperative analgesia was provided by the continuous infusion of 0.2% ropivacaine solution into the epidural space with the rate of 6–12 mL/h. The infusion was continued postoperatively.

Mean arterial pressure was maintained within 20% of the initial value, but not below 70 mm Hg. Fractional administration of phenylephrine (25–50 mg) or a continuous infusion of norepinephrine (3.2% solution) was used for the treatment of hypotension.

All patients underwent a volume-controlled mechanical ventilation with a S/5 AESPIRE (Datex-Ohmeda (GE); US) anesthetic machine (tidal volume of 6 mL/kg, respiratory rate of 10–14 per minute to maintain normocapnia, FiO_2_, 40–50%). The concept of open lung ventilation was performed as follows: after tracheal intubation the RM was performed as follows: PEEP was increased from the initial 5 to 10 cm H_2_O for 3 breaths and then PEEP was increased from 10 to 15 cm H_2_O for 3 breaths and from 15 to 20 cm H_2_O for 10 breaths and then PEEP was reduced to 12 cm H_2_O and this level was maintained till the end of the surgery [13]. This maneuver was repeated every hour. Peak (Pp) and mean (Pm) airway pressure, tidal volume, and minute ventilation were recorded. The compliance of the respiratory system (Cdyn, mL × cm H_2_O^−1^, the ratio between tidal volume and difference between Pp and PEEP) and respiratory system resistance (Raw, cm H_2_O × L^−1^ × sec^−1^) were calculated.

Prior to induction, catheterization of the radial artery was performed under the local anesthesia for the purpose of blood sampling and blood pressure measuring. Heart rate (HR, min^−1^), systolic (BPs mm Hg) and diastolic (BPd mm Hg) blood pressure, and mean arterial pressure (MAP, mm Hg.) were registered with a Nihon Kohden monitor (Japan). Stroke Index (SI, mL/m2) was determined by the transmission time of the pulse wave (esCCO technology, Nihon Kohden) with the following calculation of cardiac index (CI, l/(min/m2)) and total peripheral vascular resistance (SVR, din × s^−1^ × sm^−5^). These parameters were determined before and after the RM with the registration of maximal change. Sampling of arterial blood for analysis of the gas composition was performed 5 minutes after intubation, 5 minutes after the RM, and after extubation.

The incidence of postoperative pneumonia, the need for noninvasive ventilation, the need for intubation, hydrothorax, pneumothorax, cardiac arrest, myocardial infarction, and arrhythmias was recorded postoperatively. Registration of these complications was performed during the whole stay of patients in hospital.

Data are presented as mean (standard deviation) in a parametric distribution and median (25–75 percentile) in a nonparametric one. To assess the baseline characteristics of patients and the parameters of surgery we used Fisher's exact test for categorical variables and paired *t*-test or Wilcoxon rank test for continuous variables. For comparison of continuous variables between groups we used two-sample test or Mann–Whitney test; for dependent variables we used paired *t*-test.

## 3. Results

Both groups were comparable by age, sex, type, and duration of surgery and other characteristics (Table 1).

Applying of RMs expectedly improved oxygenation in both groups. In group H the increasing in PaO_2_/FiO_2_ ratio was on average 19% (*P* < 0,05), and, in Group M, the increasing of this value was more significant and resulted in 32% (*P* < 0,05) (Table 2). The observed increasing in the respiratory system compliance and reducing of airway resistance is expectable and specific phenomenon for the concept of open lung ventilation; there was no significant difference between the groups. Changes of respiratory biomechanics, achieved after RMs, persisted throughout the anesthesia.

On average, five RMs were performed during the anesthesia. Changes in cardiac index after RMs have been expressed in both groups, while this trend was maintained for all RMs performed during the operation. Thus, the CI decreased by an average of 18%–31% in group H compared to 18%–28% in group M (Table 3). No significant difference between groups in this case was observed.

The different dynamics was observed in SVR, which either remained unchanged or decreased by up to 14% of the initial value in group H, while, in group M, it had a tendency to increase, which was 24% of the initial value maximum (Table 4).

Such dynamics of CI and SVR led to the fact that the BP in Group H was beyond the limits of 20% of the baseline and decreased below 70 mm Hg, which did not occur in Group M (Table 5).

This fact has led to more frequent use of vasopressors (78% versus 46%, *P* < 0.05) and a greater volume of infusion (18 mL/kg/hr to 11 mL/kg/h, *P* < 0.05) in Group H compared with group M. In 23 cases in group H (versus 12 in group M, *P* < 0.05) infusion of norepinephrine was required.

No significant difference between groups in the incidence of postoperative respiratory and cardiac complications was observed. The duration of stay in the intensive care unit (3 (3–5) in Group H versus 4 (3–6) in the M group) and in the hospital (17 (10–19) in group H to 18 (12–20) in group M) did not differ between groups.

## 4. Discussion

Our data showed that recruitment maneuver improved oxygenation and respiratory biomechanics during open abdominal surgery. However, in patients with high peripheral chemoreflex sensitivity it associated with instable hemodynamics.

The revealed changes in oxygenation and biomechanical properties of the respiratory system correspond to the results of other researchers, who observed the increase in lung compliance and reduced airway resistance, as well as the increase in PaO_2_/FiO_2_ ratio [6, 17] in response to the application of the recruitment maneuver and maintaining of PEEP.

The impact of positive pressure ventilation on hemodynamic parameters is controversial. The researchers observed a complete stability of central hemodynamics after RMs even in the elderly [17] and obese patients [18]. Another part of the authors, in contrast, reported an increase in the number of used vasopressors after the RM [19, 20]. Whalen et al. reported a higher frequency of use of vasopressors (50%) in the RM group compared with the control group; however, cardiac output and mean arterial pressure were not significantly different between the two groups throughout the operation [19]. Hemmes et al. in his work also suggest a greater incidence of hypotension (46%) and a greater need for vasopressor (62%) in the RM group compared with the group without the RM (36% and 51%, resp.) [20]. Our results showed that cardiac index decreased practically in all patients, but blood pressure in patients with middle PCS remains unchanged and in this group frequency of vasopressors use was significantly lower.

The maintaining of cardiovascular system stability when applying a significant level of positive pressure in the airways depends not only on its level, but also on the functional state of the cardiorespiratory system. Fundamental studies have shown that the application of pressure of 20 cm H_2_O causes a significant decrease in SI, but at the same time, it does not have a significant impact on blood pressure due to a compensatory increase in peripheral vascular resistance [21]. However, this arrangement, as shown by the experimental model, operates only in the normal reflex regulation of the cardiorespiratory system, normal sensitivity of peripheral chemoreflex, and arterial baroreflex. When this regulation is violated, a critical reduction in the hemodynamic response to positive pressure ventilation can occur [22]. In our study, changes in central hemodynamic parameters during the RM in patients with high sensitivity of peripheral chemoreflex reflected, probably, the inability of the cardiorespiratory system to compensate for a decrease in cardiac output changes with peripheral vascular resistance, leading to the observed increase in the frequency of use of vasopressors and infusion volume.

The chosen value of breath-holding duration of 38 seconds is based on previous studies, which showed that the duration of less than 38 seconds associated with hemodynamic instability during major abdominal surgery [16]. Despite the fact that in the literature there is evidence of a strong relationship between breath-holding duration and peripheral chemoreflex sensitivity, it is practically the only work in which the cut-off point has been statistically determined.

The revealed changes in cardiac index and systemic vascular resistance are consistent with those of other researchers; however arterial baroreflex sensitivity was not determined in this study, which requires further research to clarify the safety mechanisms of hemodynamic instability during the recruitment maneuver in patients with different peripheral chemoreflex sensitivity.

## 5. Conclusion

The use of a recruitment maneuver is an effective method to improve oxygenation and biomechanical properties of the respiratory system during elective abdominal surgery. However, in patients with impaired reflex control of the cardiorespiratory system, manifested in peripheral chemoreflex hypersensitivity and decreasing of arterial baroreflex sensitivity, the recruitment maneuver associates with the risk of hemodynamic disturbances.

## Figures and Tables

**Table 1:** General characteristics of the investigated groups.

Parameter	Group H	Group M
Age, years (median (p25–p75))	69	70
	(68–78)	(66–76)
Sex (% male)	57	56
Body mass index, kg/m^2^ (mean (SD))	25	27
	(4,3)	(4,9)
Coronary heart disease (number of patients)	12	16
Hypertensive heart disease (number of patients)	28	39
Diabetes mellitus (number of patients)	3	5
Duration of surgery, min (mean (SD))	245 (65)	230 (55)
Frequency of vasopressors requirement	78%^*∗*^	46%

H: high peripheral chemoreflex sensitivity and M: middle peripheral chemoreflex sensitivity.

^*∗*^ *P* < 0,05.

**Table 2:** Parameters of oxygenation and respiratory biomechanics.

Parameter	Group	After intubation	1st hour	2nd hour	3rd hour	4th hour
PаO_2_/FiO_2_	H	287 (44)	343^*∗*^ (41)	321^*∗*^ (59)	323 (61)	333^*∗*^ (54)
	M	276 (46)	364^*∗*^ (49)	354^*∗*^ (65)	347^*∗*^ (50)	348^*∗*^ (50)
Cdyn, mL × cm H_2_O^−1^	H	63 (15)	84^*∗*^ (20)	84^*∗*^ (15)	80^*∗*^ (26)	79^*∗*^ (21)
	M	61 (16)	81^*∗*^ (22)	82^*∗*^ (21)	79 (23)	88^*∗*^ (23)
Raw, cm H_2_O × L^−1^ × sec^−1^	H	6,5 (1,8)	7,2 (2,5)	8^*∗*^ (2,4)	9,1^*∗*^ (3,1)	9^*∗*^ (2,8)
	M	6,3 (2,2)	7,4 (2,4)	7,7^*∗*^ (2,3)	8,7^*∗*^ (3,2)	8,8^*∗*^ (2,5)

H: high peripheral chemoreflex sensitivity and M: middle peripheral chemoreflex sensitivity.

^*∗*^ *P* < 0.05 versus initial value c.

**Table 3:** Dynamics of cardiac index (l/min/m^2^) (mean (SD)) before and after recruitment maneuver during anesthesia (1–5).

Number of RM	1		2		3		4		5	
Group	H	M	H	M	H	M	H	M	H	M
Before RM	3,7	3,4	3,8	3,6	3,5	3,9	4,1	4	3,6	3,9
	(0,4)	(0,35)	(0,35)	(0,25)	(0,15)	(0,3)	(0,4)	(0,25)	(0,32)	(0,36)
After RM	2,9^*∗*^	2,8^*∗*^	2,6^*∗*^	2,8^*∗*^	2,9^*∗*^	3,1^*∗*^	3,2^*∗*^	3,3^*∗*^	2,9^*∗*^	2,8^*∗*^
	(0,35)	(0,26)	(0,29)	(0,32)	(0,17)	(0,32)	(0,37)	(0,21)	(0,15)	(0,23)

H: high peripheral chemoreflex sensitivity and M: middle peripheral chemoreflex sensitivity.

^*∗*^ *P* < 0.05 compared to CI before RM.

**Table 4:** Dynamics of total peripheral vascular resistance (din × s^−1^  × sm^−5^) (mean (SD)) before and after the recruitment maneuver during anesthesia (1–5).

Number of RM	1		2		3		4		5	
Group	H	M	H	M	H	M	H	M	H	M
Before RM	1340	1240	1310	1270	1250	1170	1190	1230	1290	1180
	(50)	(90)	(110)	(100)	(90)	(80)	(120)	(90)	(75)	(95)
After RM	1150^*∗*^	1480^*∗*#^	1180^*∗*^	1440^#^	1180	1340^*∗*#^	1180	1380^*∗*#^	1270	1470^*∗*#^
	(120)	(60)	(75)	(80)	(110)	(80)	(90)	(110)	(120)	(115)

H: high peripheral chemoreflex sensitivity and M: middle peripheral chemoreflex sensitivity.

^*∗*^ *P* < 0.05 compared to SVR before RM.

^#^ *P* < 0.05 compared to group H.

**Table 5:** Dynamics of mean arterial pressure (mm Hg) (Mean (SD)) before and after recruitment maneuver during anesthesia (1–5).

Number of RM	1		2		3		4		5	
	Before RM	After RM	Before RM	After RM	Before RM	After RM	Before RM	After RM	Before RM	After RM
Group H	85	64^*∗*#^	84	72^*∗*#^	81	68^#^	87	72^*∗*#^	89	75^*∗*#^
	(12)	(9)	(12)	(8)	(9)	(7)	(11)	(12)	(14)	(9)
Group M	87	80^#^	78	72	88	85^#^	78	74	76	71
	(13)	(11)	(9)	(13)	(14)	(8)	(11)	(10)	(12)	(8)

H: high peripheral chemoreflex sensitivity and M: middle peripheral chemoreflex sensitivity.

^*∗*^ *P* < 0.05 compared to MAP before RM.

^#^ *P* < 0.05 compared to group H.
